# Comparison of anti-mullerian hormone level in non-endometriotic benign ovarian cyst before and after laparoscopic cystectomy

**Published:** 2015-03

**Authors:** Sedigheh Amooee, Mahboubeh Gharib, Parsa Ravanfar

**Affiliations:** 1 *Department of Obstetrics and Gynecology, Shahid Faghihi Hospital, Shiraz University of Medical Sciences, Shiraz, Iran.*; 2 *Shiraz Medical School, Shiraz University of Medical Sciences, Shiraz, Iran.*

**Keywords:** *Dermoid cyst*, *Ovarian cyst*, *Anti-mullerian hormone*, *Cystadenoma*, *Mucinous*, *Serous*

## Abstract

**Background:**

Benign ovarian cysts are common among both pre- and postmenstrual women. Surgical intervention for excision of an ovarian cyst is mandated when symptomatic, or chance for malignancy is high. The damaging effect of surgical ovarian cystectomy on ovarian reserve is debated in recent studies.

**Objective:**

In the present study we investigated serum level of anti-mullerian hormone (AMH) as an indicator of ovarian reserve before and after surgical cystectomy.

**Materials and Methods:**

60 patients with dermoid cyst, serous cystadenoma, and mucinous cystadenoma were recruited. Measurement of serum AMH was performed prior to surgery, and at one and 3 months after laparoscopic cystectomy. Serum AMH levels were compared before and after the surgery and between various types of ovarian cyst.

**Results:**

Serum AMH level declined significantly after the surgery which recovered to 65% of its baseline value three months later.

**Conclusion:**

Decreased serum AMH can be contributed to decreased ovarian reserve after laparoscopic ovarian cystectomy. This can result from thermo-coagulation used for hemostasis during the operation.

## Introduction

An ovarian cyst is a fluid containing sac that is formed in the ovaries. In the United States, almost all of the women in reproductive age and about 18 percent of those who are in post-menopausal age have ovarian cysts (1, 2). Most of the ovarian cysts are benign in nature with very low chance of malignant transformation. Most common symptoms of these cysts such as chronic pelvic pain, dyspareunia, gastrointestinal and urinary symptoms are resulted from the pressure they apply to the nearby structures such as the GI tract and bladder (3, 4). Management of these cysts is mandated when there is chance for malignancy (cysts larger than 4 cm and abnormal serum CA-125 levels) and intolerable symptoms. Surgical removal of the cysts is required for persistent ones measuring 5-10 cm which have not shown any shrinkage after expectant management for several cycles should be considered (5). 

The operation of resecting an ovarian cyst (ovarian cystectomy) can be performed either by laparotomy or laparoscopically. Recent studies have reported significant decrease in ovarian reserve, estimated by measurement of serum anti-mullerian hormone (AMH) levels drops significantly after ovarian cystectomy. This reduction was partially reversible three months after operation. Some other studies have reported no decrease in the serum level of AMH or damage to ovarian reserve after ovarian cystectomy (6, 7). In the present study we investigated the effect of laparoscopic ovarian cystectomy on serum AMH in patients with three most common types of ovarian cysts (Dermoid cyst, serous and mucinous cystadenoma) in Shiraz Zeinab Hospital, an affiliated center to Shiraz University of Medical Sciences.

## Materials and methods

In a Controlled before-and-after study performed in Zeinab obstetrics and gynecology hospital Hospital, affiliated to Shiraz University of Medical Sciences between Aug 2012 and Aug 2013, all 15-42 year-old women with diagnosis of benign ovarian cyst who underwent laparoscopic ovarian cystectomy were included. Before enrollment, all participants were requested to sign informed consent forms. Study protocol was approved by the ethics committee of Shiraz University of Medical Sciences.

Diagnosis of ovarian cyst was made by ultrasound imaging. Cases of benign ovarian cyst including dermoid cyst, mucinous and serous cystadenoma were included. Participants were followed by scheduled outpatient visits and serial ultrasonography imaging. No medical treatment trial was prescribed to eliminate any possible effect of GnRH therapy on pre-operative measures of AMH levels. Those who had persistent cysts measuring 5-10 cm or intolerable symptoms of adnexal mass were candidate for laparoscopic ovarian cystectomy and were included in this study. Exclusion criteria were as follows:

1. History of suspected or proved ovarian malignancy

2. Previous adnexal surgery

3. Polycystic ovarian syndrome

4. Evidence of premature ovarian failure or premature menopause

5. History of infertility or abortion

Participants scheduled for laparoscopic ovarian cystectomy were examined and serum level of AMH was measured before the operation. Preoperative and postoperative follow up ultrasound evaluations were performed by the same gynecology resident using ultrasound device (7.5 MHz trans-vaginal probe, Ultasonix OP machine; British Columbia, Canada). Pre and postoperative measurements of AMH was performed by DSL active mullerian inhibiting substance/ AMH ELISA kit (Diagnostic Systems Laboratories, Webster, Texas, United States) the same reference lab and reported in ng/ml values and detection limit of 0.006 ng/ml.

Participants underwent laparoscopic ovarian cystectomy under general anesthesia. Using a Verres needle passed through a 1cm umbilical incision, pneumoperitoneum was induced by CO_2_ insufflation to maintain an intra-abdominal pressure of 12 mmHg. Umbilical 10mm trocar and telescope was introduced which guided insertion of 3 additional 5mm trocars through supra-inguinal incisions. After exploration of pelvic cavity and visualization of ovarian cysts, antimesenteric surface of the cysts was incised using scissors. After identification of the cyst wall, it was detached from the ovarian cortex by traction and counter-traction forces applied by a traumatic grasping forceps. 

Every effort was made to excise the entire cyst avoiding the spillage of contents into the peritoneal space especially for dermoid cysts; cystectomy was performed cautiously using impermeable endobags. Bipolar cauterization and suturing was alternatively used for bleeding control. Re-approximation of incision borders was performed by fine suturing technique. After the removal of ovarian tumor, specimens were assessed by visual examination for any evidence of malignancy such as vegetation. Specimens were sent to pathology laboratory for confirmation of the diagnosis and ruling out malignancy. 

After the surgery, participants were observed in hospital ward for 48 hr to avoid surgical complications or those associated with anesthesia. For all participants, operative and post-operative course was successful with no specific complication. Participants were evaluated at outpatient clinic at 1 month and 3 months after the surgery for measuring serum level of AMH and ultrasound evaluation of surgical outcome and recurrence of the primary cyst.

**Statistical analysis**

Analysis was performed using descriptive tests and paired samples *t*-test, by SPSS 18 software (Statistical Package for the Social Sciences, version 18.0, SPSS Inc, Chicago, Illinois, USA). P-value<0.05 was considered significant.

## Results

The present study was conducted involving 60, women with average age of 25.8 (95% CI: 24.1-27.4) years. Ovarian cysts included in our study consisted of benign cysts including dermoid cyst (n=23), mucinous cystadenoma (n=11), and serous cystadenoma (n=26). Ovarian cyst diameters measured from 4.6-13 cm with mean size of 7.6 cm (95% CI: 7-8.2).

Statistical analysis of AMH levels in studied population before the operation showed a mean value of 3.77 ng/mL (95% CI: 1.58-5.96 ng/mL) which declined to 1.87 ng/mL (95% CI: 0.67-3.07 ng/mL) 1 month after the operation. This decline was significant with the p:<0.001. Measurement of AMH 3 months after the surgery revealed significant elevation to a mean level of 2.48 ng/mL (95% CI: 1.08-3.88 ng/mL) (p<0.001). Results showed that ovarian reserve recovered to 65% of its primary level 3 months after the surgery. We also compared the level of AMH before and after the operation within each type of ovarian cyst. The results were quite similar in all studied categories. 

As table I implies, within each type of ovarian cysts, variations (primary decline and long term elevation) of serum AMH levels were significant (p<0.001). Long term recovery of ovarian reserve after ovarian cystectomy did not vary significantly among serous and mucinous cystadenoma (p=0.48) but pairwise comparison of each type with dermoid cyst revealed significant difference in total recovery. Overall recovery was highest in mucinous cystadenoma (68%) and lowest in dermoid cysts (62%). The study population was categorized regarding age, primary cyst size and primary AMH level. Within each classifying variable, subgroups were compared statistically to reveal any significant variation of ovarian reserve after laparoscopic ovarian cystectomy.The results are demonstrated in the following table.As table III indicates, patients younger than 30 years old had greater decline in serum AMH level when compared to over 30 year-old patients. The difference of values were significant (p=0.024). 

Comparing the patients regarding the primary AMH level, we found that those with initial level of AMH greater than 5 ng/ml developed more decline in AMH levels than those with primary AMH less than 5 ng/ml after 3 months. For evaluation of primary cyst size effect on AMH variation after cystectomy patients were categorized as follows: Group 1) cyst size 40-65 mm, Group 2) cyst size 65-90 mm, Groups 3) cyst size larger than 90 mm. analysis for AMH level variation after 3 months showed significantly higher decline in AMH level in group 3 compared to group 1 (Table III). 

**Table I:** Variation of AMH before and after the surgery classified by cyst type

**Cyst type**	**Pre-operative AMH level (ng/mL)**	**AMH level 1 month post-operation(ng/mL)** *	**AMH level 3 months post-operation(ng/mL)** ^*^	**Overall recovery of ovarian reserve after 3 months**
Dermoid cyst (n:23)	4.13	1.71 (-58%)	2.57 (+ 51%)	62%
Mucinous cystadenoma (n:11)	3.21	1.82 (-43%)	2.21 (+21%)	68%
Serous cystadenoma (n:26)	3.69	2.04 (-45%)	2.46 (+21%)	67%

AMH: Anti mullerian hormone

* : Percentage of variation from previous value, P<0.001

**Table II:** Comparison of long term decline of AMH among various cyst types

**Compared groups**	**Cyst type**	**Mean percentage of AMH decline**	**p-value** *
Mucinous cystadenoma vs. dermoid cyst			
	Mucinous cystadenoma	30.5%	<0.001
	Dermoid cyst	37.2%	
Serous cystadenoma vs. dermoid cyst			
	Serous cystadenoma	31.3%	<0.001
	Dermoid cyst	37.2%	
Mucinous cystadenoma vs. serous cystadenoma			
	Mucinous cystadenoma	30.5%	0.446
	Serous cystadenoma	31.3%	

* : For independent *t*-test analysis for means

**Table III:** Comparison of ovarian reserve decline among different groups regarding age, primary cyst size and AMH level

**Classifying variable**			**Mean percentage of 3 month AMH decline**	**p-value**
Age				
	<30 y/o (n=40)		34.2%	0.024
	≥ 30 y/o (n=20)		31.8%	
Primary AMH level				
	< 5 ng/ml (n=38)		32.5%	0.020
	≥ 5 ng/ml (n=22)		35%	
Primary cyst size				
			32.5%	0.006*
	65-90mm (n=23)		33%	
	≥ 90mm (n=13)		35.8%	

* : For comparison of first and third group. Otherwise non-significant

**Figure 1:**
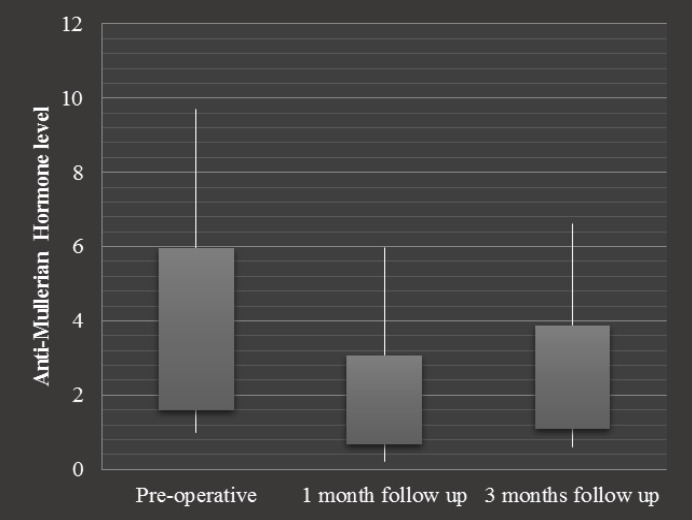
Comparison of measured serum AMH levels

## Discussion

Benign ovarian cysts are common among pre- and postmenopausal women. Ovarian cystectomy is indicated for symptomatic cysts and those at higher risk for malignancy (8, 9). Surgical intervention for resection of ovarian cyst may be performed by laparotomy or laparoscopic approach. In cases which oophorectomy is not indicated, ovarian cystectomy is performed. Ovarian cystectomy of endometriosis lesions has been associated with significant decrease in ovarian reserve assessed by measurement of serum AMH level in previous studies; especially when bipolar cauterization has been administered during the procedure. It has been shown that AMH levels recover within 3 months after the surgery but doesn’t reach the primary levels (6, 10, 11).

In the present study we aimed to evaluate the effect of laparoscopic ovarian cystectomy of four types of common benign ovarian cysts on AMH levels. We recruited 60 cases of dermoid cyst, mucinous cystadenoma, and serous cystadenoma. The population study was consisted of 23 cases of dermoid cyst, 26 cases of serous cystadenoma and 11 cases of mucinous cystadenoma. AMH levels were measured and recorded before and after the operation. Comparison of pre- and early postoperative AMH levels showed a mean percentage of 73% decrease which indicates compromised ovarian reserve and normal ovarian tissue damage. Late measurement of AMH (3 months after the surgery) revealed mild increase in AMH levels indicating degrees of recovery (mean: 32%); while complete regaining of primary ovarian reserve was not achieved (total decline in serum AMH level was 34%). 

These findings are similar to the results of the study by Chang *et al* on 20 cases mostly comprised of endometriomas (11). The surgical method of excision of ovarian cysts in the present study was similar to that of Chang *et al* in which bipolar coagulation was applied for hemostasis in the ovarian borders. This procedure is emphasized to be responsible for diminished ovarian reserve by by Litta *et al* in which authors excised 25 ovarian cysts by stripping without utilization of bipolar coagulation. Intracortical sutures were used for hemostasis to avoid thermal damage to normal ovarian parenchyma with bipolar coagulation. Measures of AMH before and after the surgery revealed no significant decrease in ovarian reserve. They concluded that the appropriate technique and avoiding administration of bipolar coagulation to healthy ovarian cortex minimizes the iatrogenic damage asserted to the ovaries during cystectomy operation (7, 11). 

We aimed to evaluate the effect of current surgical method used in our center for ovarian cystectomy (which uses bipolar coagulation to maintain hemostasis) on ovarian reserve after the surgery. Since this investigation was previously performed on mostly endometrioma cases by Chang *et al* we selected 60 cases of other ovarian cysts including mucinous and serous cystadenoma, dermoid cyst and simple ovarian cyst to eliminate any possible effect of primary cyst on the study results (7). The findings were similar among our study and the previous one by Chang *et al*. In both studies significant decrease in AMH levels was observed in the first month after operation, followed by slight recovery in the next three months to 65% of primary level. This finding is coherent with the result of Chang’s study with same surgical procedure but cannot debate the results of Litta’s study according to different surgical procedures, though we suggest performing a randomized clinical trial to determine the effect of using bipolar coagulation on ovarian reserve after laparoscopic ovarian cystectomy.

In this study we also managed to compare the degree of ovarian tissue damage among various cyst types. The results surprisingly revealed significant difference between AMH level decline in dermoid cyst and the two other studied cyst types but no variation among mucinous and serous cystadenomas was observed. Greater decrease of serum AMH levels after surgical excision of dermoid cysts which indicates greater ovarian parenchymal damage in comparison to serous and mucinous cystadenomas may lie behind the fact that the cyst walls of dermoid cysts are more adherent to underlying ovarian parenchyma and aggressive tractions for their detachment from the ovarian tissue causes greater traumatic damage to healthy parenchyma. 

Statistical analysis among two defined age groups (younger and older than 30 years) revealed significantly higher value of AMH decline in the younger age group which may correlate with higher number of primary follicles in the aged group. This fact that the density of primary follicles in each volume unit is higher in younger groups can explain that by insertion of damage to each volume unit of the high density area more follicles are likely to get injured. The same hypothesis can justify our finding regarding the impact of primary serum AMH level on the percentage of decline after the surgery. Statistical analysis showed significantly higher decrease in AMH level in the group whose primary AMH level was greater than 5 ng/ml vs. those with primary AMH level <5 ng/ml. 

The results of our study also revealed greater decline in AMH level in the group of cases whose primary ovarian cyst size was larger than 90mm versus those with 45-60 mm cysts. The effect of primary cyst size on severity of damage to ovarian reserve may be explained by the fact that larger ovarian cysts mandate more surgical manipulations for resection and also result in more bleeding for which larger areas of the ovarian border should be cauterized. In the most recently published studies, Sugita and colleagues investigated the variation of ovarian reserve in 42 cases of unilateral or bilateral endometrioma before and one year after the surgery for ovarian cystectomy. Their results were quite similar to our findings that showed immediate greater decline and long term recovery of the ovarian reserve (12). In the other most recent study conducted by Alborzi *et al* the authors recruited 193 cases of endometrioma who underwent laparoscopic cystectomy (13). 

The level of AMH was measured preoperatively and one week, three and nine months postoperatively. The pattern of initial decline and long term recovery was similar in their study to the findings of Chang *et al*, Sugita *et al *and the present study. It should be mentioned that all studies was conducted with same surgical procedure including bipolar coagulation for hemostasis whenever needed (7, 12, 13). In the study by Alborzi *et al* they also emphasized that bilateral presence of endometrioma results in greater decline in serum AMH. They also reported that older groups are susceptible to greater damage to ovarian reserve which is in contrary with our finding. To maintain hemostasis during ovarian cystectomy suturing techniques seem to be associated with less ovarian parenchymal damage than bipolar cauterization; though we suggest further studies to compare the effect of these two procedures on ovarian reserve.

## Note

***This article extracted from M.D. thesis. (Mahboubeh Gharib)***
